# Benzyl Isothiocyanate, a Major Component from the Roots of *Salvadora Persica* Is Highly Active against Gram-Negative Bacteria

**DOI:** 10.1371/journal.pone.0023045

**Published:** 2011-08-01

**Authors:** Abier Sofrata, Ellen M. Santangelo, Muhammad Azeem, Anna-Karin Borg-Karlson, Anders Gustafsson, Katrin Pütsep

**Affiliations:** 1 Department of Dental Medicine, Karolinska Institutet, Stockholm, Sweden; 2 Department of Chemistry, School of Chemical Science and Engineering, Royal Institute of Technology, Stockholm, Sweden; 3 Department of Microbiology, Tumor and Cell Biology, Karolinska Institutet, Stockholm, Sweden; Charité, Campus Benjamin Franklin, Germany

## Abstract

Plants produce a number of antimicrobial substances and the roots of the shrub *Salvadora persica* have been demonstrated to possess antimicrobial activity. Sticks from the roots of *S. persica*, Miswak sticks, have been used for centuries as a traditional method of cleaning teeth. Diverging reports on the chemical nature and antimicrobial repertoire of the chewing sticks from *S. persica* led us to explore its antibacterial properties against a panel of pathogenic or commensal bacteria and to identify the antibacterial component/s by methodical chemical characterization. *S. persica* root essential oil was prepared by steam distillation and solid-phase microextraction was used to sample volatiles released from fresh root. The active compound was identified by gas chromatography-mass spectrometry and antibacterial assays. The antibacterial compound was isolated using medium-pressure liquid chromatography. Transmission electron microscopy was used to visualize the effect on bacterial cells. The main antibacterial component of both *S. persica* root extracts and volatiles was benzyl isothiocyanate. Root extracts as well as commercial synthetic benzyl isothiocyanate exhibited rapid and strong bactericidal effect against oral pathogens involved in periodontal disease as well as against other Gram-negative bacteria, while Gram-positive bacteria mainly displayed growth inhibition or remained unaffected. The short exposure needed to obtain bactericidal effect implies that the chewing sticks and the essential oil may have a specific role in treatment of periodontal disease in reducing Gram-negative periodontal pathogens. Our results indicate the need for further investigation into the mechanism of the specific killing of Gram-negative bacteria by *S. persica* root stick extracts and its active component benzyl isothiocyanate.

## Introduction

Periodontitis is a complex disease in which inflammatory responses due to bacteria and host interactions play a major role in expression and progression of the disease [1]. Although host genetics, environmental and acquired factors are essential in the development of periodontal disease, bacterial accumulation leading to microbial challenge is the primary initiating factor [2]. Gram-negative bacteria such as *Tannerella forsythia* (*Bacteroides forsythus*), *Porphyromonas gingivalis*, and *Treponema denticola* are found in significantly higher levels in patients with chronic and aggressive periodontitis and therefore often called the red complex bacteria [3]–[5]. These Gram-negative bacteria, along with *Aggregibacter (Actinobacillus) actinomycetemcomitans*, colonize the gingival pockets; they are strong inducers of inflammation and tissue destruction and with a highly developed capability to evade host immune responses and resist antimicrobial treatment [6]–[11].

Historically, the first known oral hygiene tool was the chewing stick, the Miswak. Throughout the world, 182 species of plants have been used as sources for chewing sticks out of which the roots of the shrub *Salvadora persica* is the most common one. Its use remains widespread in many parts of the world from East Africa through to the Asian subcontinent [12]. In addition to the mechanical removal of dental plaque and stimulation of the gingival tissues, there were early reports indicating that the root of *S. persica* exerts antimicrobial activity [13]–[15]. However, these studies diverge in methods used for root preparation or extraction. Furthermore, the reported microbicidal effect of *S. persica* root water- or alcohol extracts have been modest and the antimicrobial repertoire discerned varied between the different studies due to variation in analysis methods applied [16]–[19]. We have previously demonstrated that water extracts of *S. persica* chewing sticks raise the pH of dental plaque by increasing the parotid gland secretion and thus may have an impact on plaque bacteria [20]. More recently we could establish that cut chewing stick pieces from the root of *S. persica* that were suspended by a thread above the surface of a bacterial lawn exhibited strong bacterial killing capacity [21].

The first aim of this study was to determine the antibacterial repertoire of *S. persica* root extracts and volatiles by using a panel of different bacterial species, including the two pathogens most strongly associated with periodontitis. The second aim was to identify and isolate the antibacterial component(s) using a chemically broad approach combining adsorption of volatiles, different chromatographic methods and mass-spectrometry. We found that *S. persica* extracts and volatiles contained one major antibacterial component, benzyl isothiocyanate (BITC), with rapid bactericidal effect against all Gram-negative bacteria including periodontal pathogens, but low effect on Gram-positive bacteria. Electron microscopy revealed that the bacterial envelope was severely damaged by both *S. persica* extracts as well as commercial pure BITC.

## Materials and Methods

### Steam distillation of Miswak

To obtain the essential oil of *S. persica* root, 1.5 kg of root sticks were cut into 1–2 cm pieces in length. The pieces were ground using a stone mill and thereafter mixed with 700 ml of distilled water in a distillation flask. The mixture was heated and temperature of the steam was maintained at 80°C during the distillation. Steam from an additional container with boiling water was continuously added to the distillation flask allowing the compounds to evaporate as an oil water mixture which was cooled to room temperature. The oil/water mixture obtained was extracted with HPLC grade hexane (M.I, Merck, WVR International, Sweden) and the hexane was evaporated under reduced pressure at 20°C.

### Bacterial strains and growth conditions

#### Gram-negative bacterial strains

the oral periodontal pathogens *Aggregatibacter actinomycetemcomitans* HK 1519 (provided by Dr M. Kilians, Aarhus, Denmark) and *Porphyromonas gingivalis* (ATCC 33277), the pathogenic *Salmonella enterica* serovar Typhimurium (ATCC 14028) and *Pseudomonas aeruginosa* strain PA01 and the opportunistic pathogen *Haemophilus influenzae* (ATCC 49247). *Escherichia coli* laboratory strain MC4100 and *Escherichia coli* K12 LPS-mutant strain D21f2 [22] represent commensal Gram-negative bacteria. **Gram-positive bacterial strains:** the oral pathogens *Streptococcus mutans* (CCUG 27624) and *Lactobacillus acidophilus* (NCTC 1723), the opportunistic pathogens *Streptococcus pyogenes* (clinical isolate, provided by Dr M. Lindberg, Uppsala Sweden) and *Staphylococcus aureus* strain 8325-4 (provided by Dr S. Arvidsson, Karolinska Institutet Sweden), and the commensals *Lactobacillus fermentum* (DSM 20052), *Enterococcus faecalis* (ATCC 29212) and *Enterococcus faecium* (clinical isolate provided by Dr I. Kühn, Karolinska Institutet Sweden). **Growth conditions:** *A. actinomycetemcomitans* was propagated on Colombia base agar (Acumedia, Baltimore, MD, USA) supplemented with 0.01% tryptophan (Merck, VWR International, Sweden) and citrated horse blood (5%) in a 5% CO_2_ atmosphere (CampyPak™, Becton Dickinson, Sweden). *P. gingivalis* was propagated for 6 days on Colombia base agar supplemented with hemin (0.05 mg/ml), vitamin K (0,01 mg/ml) (BBL™, Becton Dickinson, Sweden) and citrated horse blood (5%) in an anaerobic atmosphere (GasPak™, Becton Dickinson). *S. mutans* was grown on BHI (Brain Heart Infusion) agar plates (Oxoid, Malmö, Sweden) for two days in 5% CO_2_ atmosphere generated with gas-packs. *H. influenzae* was propagated over-night on GC-agar (Acumedia) supplemented with haemoglobin (1%) (BBL™) and isovitalex enrichment (1%) (BBL™) in 5% CO_2_ atmosphere generated with gas-packs. *L. acidophilus* and *L. fermentum* were propagated for two days on Lactobacilli MRS agar plates (Difco™, Becton Dickinson, Sweden) in 5% CO_2_. *S. pyogenes*, *S. aureus*, *E. faecalis*, and *E. faecium* were all propagated overnight on TSB-Y agar plates (1,5% Bacteriological agar No 1 in Tryptic Soy Broth, (Bacto™, Becton Dickinson, Sweden) supplemented with 0.5% yeast extract (BBL™) in normal atmosphere. *E. coli* MC4100, *E. coli* D21, *P. aeruginosa*, and *S. typhimurium* were propagated overnight in air on Luria Agar Base, Miller (Difco™).

### Antibacterial dose-response assay

Colonies of *A. actinomycetemcomitans* or *P. gingivalis* were resuspended in Peptone Yeast Glucose medium (PYG). *S. mutans* colonies were resuspended in BHI broth (Oxoid) and *H. influenzae* colonies were resuspended in 5% CO_2_ preconditioned BHI medium with 5% Fildes Enrichment (BBL™). *L. acidophilus* and *L. fermentum* colonies were resuspended in Lactobacilli MRS Broth and *S. pyogenes*, *S. aureus*, *E. faecalis*, and *E. faecium* were all resuspended in Tryptic Soy Broth (TSB) (Bacto™) supplemented with 0.5% yeast extract for antibacterial assays. In all cases the bacteria colonies were resuspended to an optical density of 0.5 at the wavelength λ_590 nm_. *E. coli* MC4100, *E. coli* D21, *P. aeruginosa*, and *S. typhimurium* were grown to the same optical density as above in Luria Broth (Difco™). All bacteria were further diluted in fresh growth medium 10^4^ fold prior to the test. The bacterial suspensions were incubated for 90 minutes in their respective growth medium at 37°C in the presence of different concentrations of essential oil. Dilutions of the oil were prepared in dimethyl sulfoxide (DMSO) (D2650, Sigma-Aldrich, Sweden) and five µl of each dilution or undiluted oil was added to 500 µl bacteria suspension to obtain final concentrations of 1%, 0.1% 0.05%, 0.02%, 0.01%, 0.005% and 0.001% oil. To the controls 5 µl of DMSO was added. After 90 minutes, following serial dilutions, the suspensions were spread on agar-plates with growth medium. The plates were incubated at 37°C degrees until bacterial colonies were visible. The number of live bacteria was determined by counting the number of colonies on each plate, which equals the number of the colony forming units (CFU). Each CFU-determination was performed in duplicates and for each bacterium and oil concentration the experiment was repeated three times. The pooled MPLC-fractions from extracted *S. persica* root were analyzed for antimicrobial activity against *E. coli, L. acidophilus, S. pyogenes* and *A. actinimycemcomitans* using the CFU-assay and the experiment was repeated twice.

### Bacterial killing kinetics

The number of viable *A. actinomycetemcomitans* and *E. coli* MC 4100, respectively, were assessed after co-incubation with essential oil in a final dilution of 0.1% oil for a time-period of 2, 5, 10, 20, 40 or 90 minutes. Aliquots were removed after each time point and plated for determination of CFU. The CFU determination was performed as above and the experiment was repeated three times.

### Solid-phase microextraction (SPME) of root stick volatiles

One piece of Miswak stick (2.0 cm) was placed in a 50 ml beaker. A solid-phase microextraction (SPME) needle was inserted into the beaker through a pin hole in the aluminum foil and volatiles from the cut root were collected for 15 minutes using a SPME- fibre coated with the polymer polydimethylsiloxane/divinylbenzene (Supelco, Sigma-Aldrich Sweden). Volatiles from the cut root were collected for 15 minutes. The compounds adsorbed on the SPME fibre were desorbed in a GC-MS injector and identified by gas chromatography-mass spectrometry (GC-MS), see below. The collection of volatiles and analysis were repeated three times.

### Medium-Pressure Liquid Chromatography

The preparative medium-pressure liquid chromatography (MPLC) technique of combined extraction and chromatography was used for isolation of compounds with a wide range in polarity and boiling points [23]. Fresh Miswak sticks were cut into 1 cm pieces and minced with silica (Merck 60, 230–400 mesh (0.040–0.063 mm) Carlo Erba Reactifs, SDS France, Chemtronica AB Sweden) by means of a household meat grinder (Moulinex, France) in a 1∶1 mixture (50.4 g silica +50.4 g Miswak). The material was passed through the grinder four times to produce a free flowing powder. The powder was dry packed into a 30 mm internal diameter glass column (Baeckström Separo AB, Sweden) followed by fresh silica gel (18.4 g). The length of the loading zone was 19 cm and that of the fresh silica was 5.6 cm. The column was sealed with pistons that could be adjusted to the resulting bed length. Two consecutive gradients were used for elution. The first gradient was accomplished by using 100 ml of pure hexane (M. I., HPLC grade, Carlo Erba Reactifs-SDS) in the mixing chamber followed by consecutively adding 100 ml of mixtures of hexane and ethyl acetate (99.8% HPLC grade, Carlo Erba Reactifs) of 1.25, 2.5, 5, 10, 20, 40, and 80% ethyl acetate in hexane and finally with 300 ml 100% ethyl acetate. This was followed by consecutively adding 100 ml of mixtures of methanol (99.8%, HPLC grade, Carlo Erba Reactifs) in ethyl acetate (1.25 5, 10, 20, 40, and 80%), ending with 300 ml of 100% methanol. Altogether, 70 MPLC-fractions of 20 ml were collected throughout the separation and elution procedure.

### Thin-layer chromatography

The MPLC fractions were analyzed by thin-layer chromatography (TLC), on silica gel 60 F_254_ (Merck, VWR international, Sweden) pre-coated aluminium sheets. Elution was employed with increasing polarity by 10% ethyl acetate in hexane, 20% ethyl acetate in hexane and 30% methanol in ethyl acetate, respectively. For staining the TLC plates were sprayed with a solution containing 3 g vanillin (99% Lancaster synthesis, Chemtronica AB, Sweden) and 0.5 ml sulphuric acid (99.99%, Sigma Aldrich Sweden) dissolved in ethanol (99.5%, Kemetyl, Sweden) and then developed by heating. On the basis of similar retardation factors (Rf) the MPLC-fractions were pooled into eight samples with increasing polarity. These eight samples were subjected to a second TLC-analysis and the amount of BITC in every sample was determined (see below). The eight samples were then evaporated under reduced pressure at 20°C and re-dissolved in DMSO (99.5%, Merck, VWR International, Sweden) to test for antibacterial antimicrobial activity against *E. coli, L. acidophilus, S. pyogenes* and *A. actinimycemcomitans* using the CFU-assay. For identification by GC-MS the samples were dissolved in Hexane/EtOAc (9/1 v/v).

### Gas chromatography-mass spectrometry

Gas chromatography-mass spectrometry (GC-MS) was performed using a Varian 3400 GC connected to a Finnigan SSQ 7000 quadropole mass spectrometer. The GC was equipped with a split/splitless injector (splitless mode 30 sec), a DB-wax capillary column (30 m, 0.25 mm inner diameter and 25 µm film thickness) J & W Scientific USA. Injector temperature was isothermally set at 230°C. Carrier gas (Helium, 99.99%, Stransmöllen AB Sweden) was delivered at a constant pressure of 10 psi. A representative temperature programme was 40°C for 1 minute, followed by an increase in temperature with 4°C/min up to 235°C, and then the temperature was maintained at 235°C for 10 minutes. Transfer line temperature was kept at 240°C and the MS ion source temperature was 150°C. Mass spectra were obtained at 70 eV with a mass range of 30 to 600 m/z in positive mode.

Pooled MPLC fractions and essential oil samples for GC-MS analysis were reconstituted in hexane by dissolving 10 mg of each completely dried sample in 0.9 ml of hexane and 0.1 ml ethyl acetate. The samples were further diluted to 2 mg/ml and 1 µl was injected for the analysis. For the analysis of the volatiles from the cut Miswak stick the SPME fibre was desorbed in the GC injector at 225°C, for 4 minutes. The compounds eluted faster due to a different GC programming than in the analyses of the essential oils and fractions. The constituents of the samples were identified by comparison with Finnigan NIST library and confirmed by analysing the reference compounds using the same chromatographic parameters.

For the quantitative determination of benzyl isothiocyante (BITC) in the pooled MPLC fractions and the essential oil, a calibration curve was created using the synthetic BITC (>98% GC-purity, Lancaster Synthesis Inc., Windham, NH. USA). Five concentrations of synthetic BITC (0.00547 µg, 0.0218 µg, 0.0875 µg, 0.35 µg and 1.4 µg) were injected into the GC-MS under the same conditions as for the MPLC pooled fractions and essential oil samples. A calibration curve was obtained with Y = 4.7495E7+1.7387E9*x and R2 = 0.9953.

### Transmission electron microscopy

The Gram-negative bacterium *A*. *actinomycetemcomitans* was incubated for 2, 20 or 40 minutes at 37°C with 0.1% of essential oil (corresponding to 2.8 µmol BITC). The control was of a final concentration of 1% DMSO (99.5%, Merck, VWR International, Sweden). For comparison, 1 mg/ml of the antibiotic Ampicillin (Ampicillin sodium salt, A9518, Sigma-Aldrich, Sweden) or 2.8 µmol of synthetic BITC were assessed. At each time point an aliquot of bacterial suspension was removed and centrifuged for 10 min. The supernatant was discarded and bacteria were suspended in fixation buffer containing 2% glutaraldehyde (Ladd, Burlington, Vermont, USA) in 0.1 M sodium cacodylate buffer (Merck, Darmstadt, Germany) containing 0.1 M sucrose (Merck, Darmstadt) and 3 mM CaCl_2_ (Merck, Darmstadt) pH 7.4 at 4°C overnight. Following fixation the bacteria were centrifuged to a pellet. The pellet was rinsed in 0.1 M phosphate buffer (Merck, Darmstadt) pH 7.4 followed by post-fixation in 2% osmium tetroxide (TAAB, Berkshire, England) in 0.1 M phosphate buffer, pH 7.4 at 4°C for 2 h. The fixated samples were dehydrated in ethanol followed by acetone and embedded in LX-112 (Ladd, Burlington). Sections were contrasted with uranyl acetate (Ladd, Burlington) followed by lead citrate (Sigma-Adrich, Sweden) and examined in a Leo 906 transmission electron microscope (Zeiss, Oberkochen, Germany) at 80 kV. Digital images were taken by using a Morada digital camera (Soft Imaging System, GmbH, Münster, Germany).

## Results

### Preparation of *S. persica* essential oil by steam distillation

To characterize the antibacterial properties of *S. persica* root chewing sticks we extracted essential oil from milled sticks by steam distillation. Steam was added during distillation allowing the compounds to evaporate at lower temperatures to avoid loss of active material. Steam distillation of 1.5 kg of *S. persica* root sticks yielded 12 g of essential oil.

### Assessment of antibacterial properties of *S. persica* essential oil

**Figure 1:**
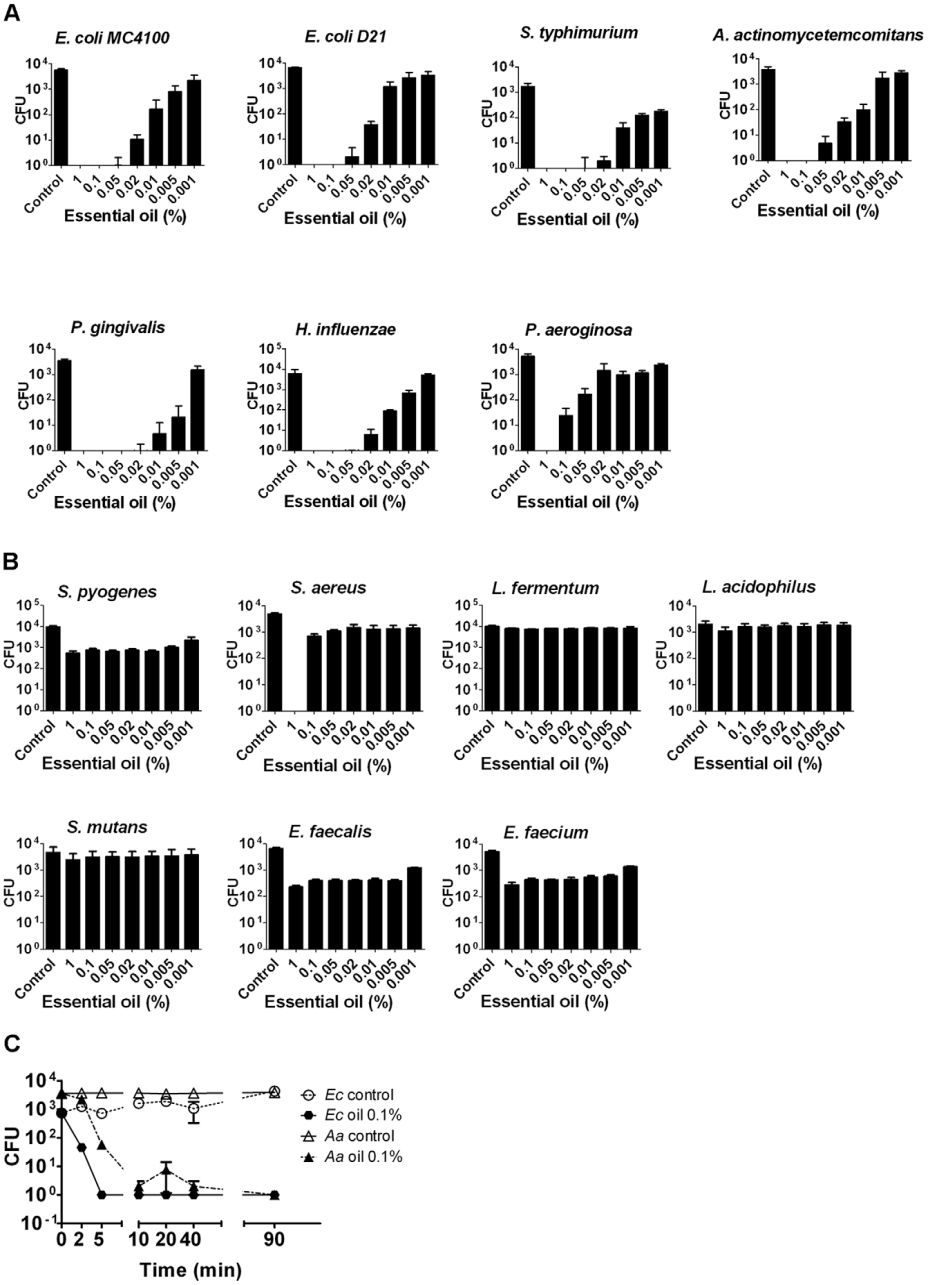
Antibacterial activity of *S. persica* essential oil determined by colony forming units (CFU) assay. The individual bars show the statistical mean and standard deviation of the number of surviving bacteria from three experiments. The essential oil concentration is in percentage of final assay volume. Control is with DMSO only. (A) Gram-negative bacteria (B) Gram-positive bacteria (C) Kinetics of antibacterial activity against *E. coli* MC4100 (Ec) and *A. actinomycetemcomitans* (Aa) Samples were withdrawn for CFU determination at time-points indicated. Oil dilution was 0.1%. The CFU values displayed are the statistical mean of three experiments.

To assess the antibacterial properties of the essential oil the following bacteria were used; the oral pathogens *A. actinomycetemcomitans, P. gingivalis, S. mutans* and *L. acidophilus,* the intestinal pathogen *S. typhimurium,* the environmentally acquired pathogen *P. aeruginosa*, and the opportunistic pathogens *S. aureus, S. pyogenes, H. influenzae* as well as the commensals *E. coli, E. faecalis, E. faecium, L. fermentum*. The LPS mutant of *E. coli* K12 strain D21f2 [22] was included due to its higher susceptibility to antibiotics. The essential oil exhibited high antibacterial activity in a dose-dependent manner against the Gram-negative bacteria while the Gram-positive bacteria were essentially unaffected or displayed limited growth inhibition (Figure 1A and Figure 1B). *S. aureus* was killed at the concentration of 1% oil, while being resistant to killing by further oil dilutions. This threshold effect was reproducible but not observed for any of the other Gram-positive bacteria tested.

### Kinetics of bacterial killing

Two BITC-susceptible bacteria, the oral periodontal pathogen *A. actinomycetemcomitans* and the *E. coli* laboratory strain MC 4100, were chosen in order to determine the kinetics of bacterial killing. We used the 0.1% essential oil concentration to appreciate the kinetics extending over one bacterial generation time. The kinetics of the killing activity was rapid (Figure 1C). A pronounced bactericidal effect was already apparent at 5 minutes and a 1000-fold reduction of surviving bacteria was recorded after 10 minutes for both bacteria.

### Chemical characterization of volatile compounds and essential oil from Miswak

**Figure 2:**
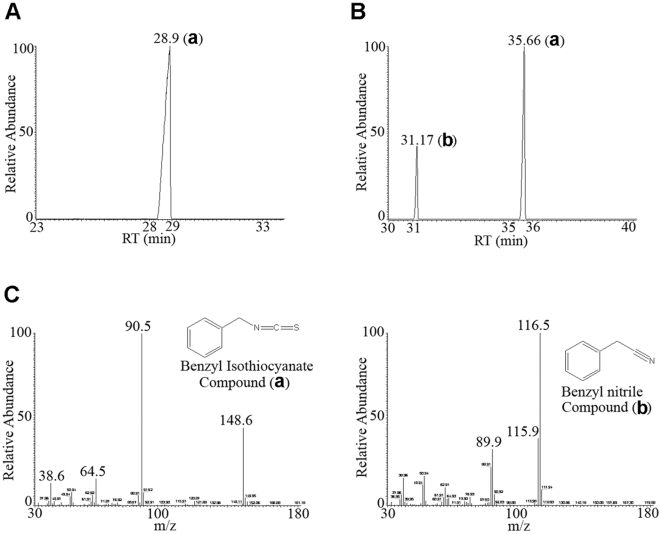
Gas chromatography-mass spectrometry (GC-MS) analysis of *S. persica* root volatiles and essential oil. (A) Chromatogram of Miswak volatiles (B) Chromatogram of Miswak essential oil. (C) Mass spectrometry (MS) analysis and identification of compound a as benzyl isothiocyanate MW = 148.6 (theoretical mass 149.21) and compound b as benzyl nitrile, MW = 116.5 (theoretical mass 117.15). The chemical structures are presented as insets in the figure.

Solid-phase microextraction (SPME) was used for collection of the volatile bactericidal components from *S. persica* root-pieces. GC-MS of the collected volatiles revealed that the major component, >98%, was benzyl isothiocyanate (BITC), corresponding to compound (a) (Figure 2A and Figure 2C).

GC-MS analysis of the essential oil revealed two components, with the distribution of 73.8% benzyl isothiocyanate (compound a) and 26.2% benzyl nitrile (compound b) (Figure 2B and Figure 2C). Benzyl isothiocyanate is, thus, the dominant component of *S. persica* root essential oil.

### Assessment of synthetic BITC bactericidal activity

**Figure 3:**
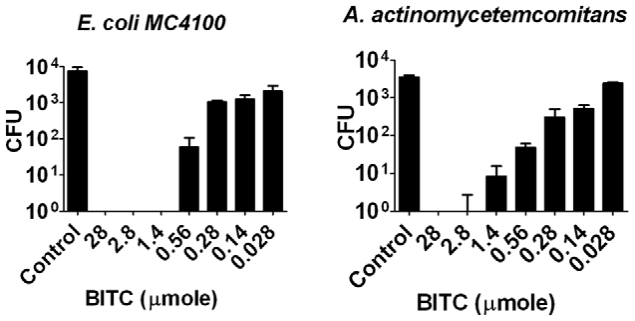
Antibacterial activity of commercial synthetic BITC against two Gram-negative bacteria. The individual bars show the statistical mean and standard deviation of the number of surviving bacteria from three experiments. The amount of BITC applied corresponds to the BITC content in the different oil dilutions of figure 1; 28 µmole (1% oil), 2.8 µmole (0.1% oil), 1.4 µmole (0.05% oil), 0.56 µmole (0.02% oil), 0.28 µmole (0.01% oil), 0.14 µmole (0.005% oil) and 0.028 µmole (0.001% oil) pure BITC, respectively. Control is with DMSO only.

The content of BITC in the essential oil was 73.8%. The amount of BITC in the different oil dilutions used in Figure 1 corresponds to 28 µmol, 2.8 µmol, 1.4 µmol, 0.56 µmol, 0.28 µmol, 0.14 µmol and 0.028 µmol, respectively. Synthetic BITC used at the same concentration as it occurs in the oil was thus as efficient against the bacteria *A. actinomycetemcomitans* and *E. coli* (Figure 3) as was the *S. persica* root essential oil (Figure 1A).

### Characterization of *S. persica* root constituents by MPLC

**Figure 4:**
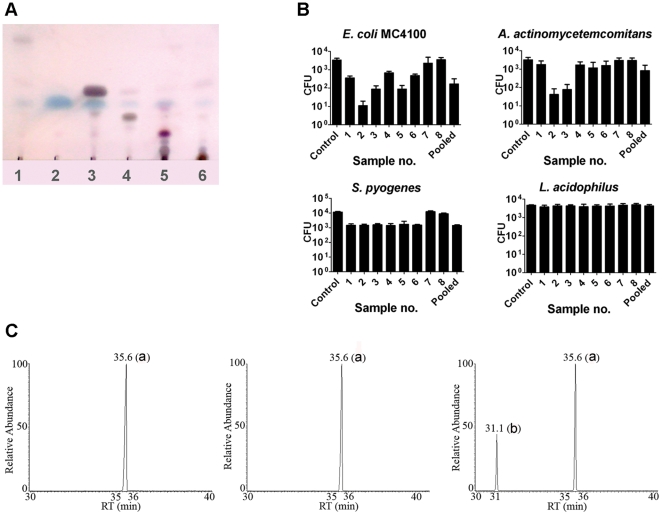
Analysis of *S persica* root components by thin layer chromatography, antibacterial activity and GC-MS. (A) TLC analysis of MPLC extracted-fractionated samples 1–6. Samples 7 and 8 are not shown since their components were too hydrophilic to be mobile in the ethyl acetate-hexane solvent. (B) Antibacterial activity of MPLC samples against two Gram-negative and two Gram-positive bacteria. The individual bars show the statistical mean and standard deviation of the number of surviving bacteria from two experiments. Pooled, denotes samples 1–8 mixed together. Control is with DMSO only. (C) Chromatogram of MPLC-samples with antibacterial activity. Sample 2 (left) and sample 3 (middle) displayed one major compound (a) = benzyl isothiocyanate. Sample 5 (right) displayed both compound (a) and compound (b) = Benzyl nitrile.

Combined extraction and chromatography, using MPLC, was performed in order to evaluate the possible contribution to the antibacterial activity of constituents with a broad range in polarity. The MPLC extraction-chromatography yielded 70 fractions which were analyzed with TLC (Figure S1) and fractions containing compounds with a similar retardation factor were pooled, resulting in eight MPLC-samples (Table S1). These eight samples were subjected to a second TLC-analysis (Figure 4A) and the amount of BITC in every sample was determined (Table 1) by GC-MS analysis and calculated from the calibration curve. Sample number 1–6 displayed antibacterial activity while sample 7 and 8, which were more polar and did not display any mobility in the TLC (Figure 4A), had essentially no impact on bacterial viability as compared to the DMSO control (Figure 4B). Samples 2 and 3 displayed the highest antibacterial activity as well as the highest concentration of BITC (Table 1 and Figure 4B). The antibacterial repertoire of these two MPLC-samples was similar to that of *S. persica* essential oil in displaying killing activity against Gram-negative bacteria (Figure 1A, Figure 1B, and Figure 4B). Finally, all eight MPLC-samples were pooled together in order to test for lost synergy between separated components. This pooled sample displayed no higher antibacterial capacity compared to any of the most active single samples, thus underscoring that BITC is the major antibacterial component of *S. persica* root sticks (Figure 4B). The GC-MS analysis of samples 2, 3 and 5 (Figure 4C) confirms that BITC is a major component in samples with the highest antibacterial activity.

**Table 1:** Benzyl isothiocyanate (BITC) in pooled MPLC-samples.

MPLC sample	Sample weight (g)	BITC (%)
1	0.014	6.57
2	0.234	89.43
3	0.029	72.23
4	0.039	5.20
5	0.044	22.97
6	0.080	6.11
7	0.117	0
8	2.721	0

### Visualization of essential oil effects on bacteria

**Figure 5:**
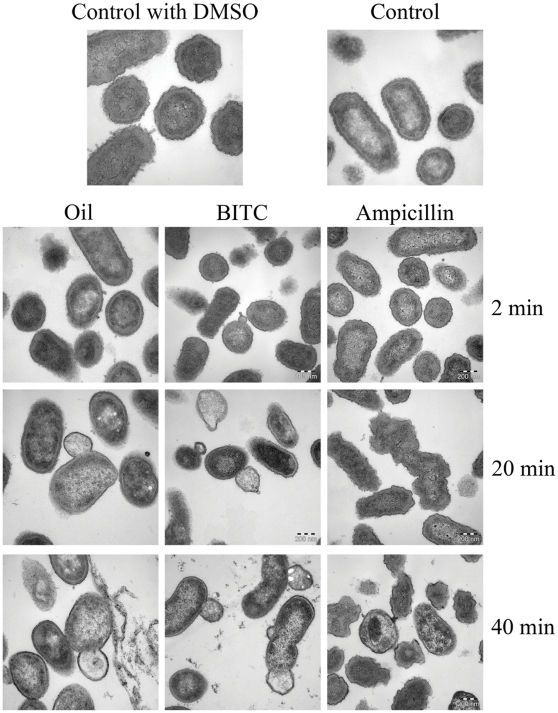
Transmission electron micrographs of *A. actinomycetemcomitans* treated with essential oil, BITC or the antibiotic ampicillin. Samples were withdrawn at time points indicated. Concentration of essential oil was 0.1%, which corresponds to a content of 2.8 µmole BITC. The amount added synthetic BITC was 2.8 µmole and ampicillin concentration was 1 mg/ml. Control for essential oil and BITC contained 1% DMSO. For ampicillin, the control was with 1% water. The control bacteria were withdrawn after 40 min of incubation.

In order to understand how the *S. persica* root essential oil affects the bacteria we used transmission electron microscopy. We chose the susceptible periodontal pathogen *A. actinomycetemcomitans* and the electron micrographs of the oil-treated bacteria revealed dramatic effects on the cell membrane. In the presence of the essential oil the bacterial membrane exhibited small protrusions after two minutes of incubation (Figure 5). These protrusions increased in size and prevalence with increasing incubation time leading to loss of bacterial membrane integrity. The commercially available pure BITC induced similar protrusions from the bacterial membrane. The antibiotic ampicillin, which was used as a reference antibiotic that targets cell-wall synthesis, did not give rise to bacterial cell membrane protrusion.

## Discussion

The transition from periodontal health or gingivitis to periodontitis is characterized by a shift from a predominantly Gram-positive microflora to a more Gram-negatively dominated flora [5], [24]. Our finding that extracts of chewing sticks, Miswak, from the *Salvadora persica* root target Gram-negative bacteria indicates that these chewing sticks may be useful in maintaining or restoring a microflora associated with periodontal health and stability. Previous reports on the antimicrobial capacity of *S. persica* root sticks are not concurrent, when it comes to their antibacterial repertoire. Herein we demonstrate that *S. persica* root essential oil killed the Gram-negative oral pathogens *A. actinomycetemcomitans* and *P. gingivalis*. Moreover, all the other Gram-negative bacteria were killed as well, including medically important Gram-negative pathogens, such as *Haemophilus influenzae* and *Salmonella enterica*. This indicates that the killing was specific to Gram-negative bacteria rather than related to the life-style acquired by periodontal pathogens. The kinetics of bacterial killing was rapid. The bacterial load diminished a 1000-fold within minutes. Contrary to the strong bactericidal effect on Gram-negative bacteria, the Gram-positive bacteria, including the Gram-positive oral pathogens *S. mutans* and *L. acidophilus*, were essentially unaffected by the oil or displayed only growth inhibition. The short exposure-time required for the bactericidal effect against Gram-negative bacteria to occur supports the notion that chewing-sticks from *S. persica* may be efficient in improving periodontal health since the main periodontal pathogens are Gram-negative. There are only a few studies evaluating the *in vivo* effects of the practice of chewing sticks from *S. persica* on oral health and the conclusions vary with the study design [25], [26]. Larger epidemiological studies with DNA-based analysis of the oral microbiota would be necessary in order to further evaluate the efficacy of *S. persica* chewing sticks in daily oral hygiene.

To apply a broad chemical approach for identifying the bioactive compounds from the root of *S. persica*, we used three methods to extract the antibacterial compounds. Firstly, we determined by SPME and GC-MS that the highly antibacterial volatiles from *S. persica* root sticks consist to more than 98% of benzyl isothiocyanate (BITC). Secondly, the essential oil obtained by steam distillation revealed two components, BITC (73.8 %) and benzyl nitrile (26.2%), which is similar to what was found in an early study of *S. persica* essential oil [27]. Finally, to examine whether other components in the plant root besides BITC could confer bactericidal activity, especially compounds with higher molecular weight or more polar compounds,we used a combined preparative extraction-fractionation technique by medium-pressure liquid chromatography (MPLC). Those fractions that contained BITC as a dominant component exhibited the highest bacterial killing capacity. BITC is thus, the main bactericidal factor of *S. persica* root sticks, active both at a distance as a volatile and in extracted form against Gram-negative bacteria. It is noteworthy that the essential oil from *S. persica* root was remarkably devoid of additional hydrophobic components as compared to the terpene-rich essential oil from its leaves [28]. Possibly, there could be other polar components in the root sticks that can be obtained through water or alcohol extraction that may contribute to the antibacterial activity. However, the highly polar fractions from *S. persica* root sticks in our study did not exhibit any antibacterial activity.

Similar to the case with *S. persica* root extracts, the previously reported antibacterial repertoire of pure BITC differs in between studies [29]–[31]. These differences may be due to variations in methods used for antibacterial testing. Herein we used bacteria in suspension in order to avoid any inhibitory effects of agar in bacterial susceptibility tests [21], [32]. In an early study, Al-Bagieh and Weinberg used bacteria in suspension and reported growth inhibitory effect of BITC on the caries-associated Gram-positive bacterium *Streptococcus mutans* [30]. However, since no Gram-negative bacteria were tested the strong bactericidal effect of BITC against Gram-negative bacteria remained unnoticed.

The rapid bacterial killing implied that *S. persica* root stick extract might target the bacterial membrane. Electron micrographs of the Gram-negative bacterium *A. actinomycetemcomitans* revealed, that both essential oil as well as pure commercial BITC gives rise to similar protrusions in the bacterial cell membrane. The membrane protrusions resemble those formed under the influence of antimicrobial peptides [33]. Some of these peptides act through insertion into the bacterial membrane, which leads to lost membrane potential and membrane integrity [34]. However, as BITC has both lipophilic as well as electrophilic properties we speculate that BITC might penetrate through the outer bacterial membrane and possibly interfere with the bacterial redox systems and thus hamper the ability of the bacterium to maintain its membrane potential. Such an effect of BITC has been demonstrated for mitochondrial membranes [35].

BITC is an effector molecule of the defence system of many cruciferous plants such as cabbage, watercress and broccoli [36]. Upon plant tissue damage, BITC is released by hydrolysis of the compound benzyl-glucosinolate through the action of the enzyme myrosinase [37], [38]. The requirement for enzymatic processing to obtain antimicrobial effect is in line with our observation that boiled sticks lose their antibacterial activity (unpublished data). This enzyme-mediated release of BITC occurs also when a *S. persica* chewing stick is chewed on prior to cleansing of teeth. The chewing gives rise to a brush-like structure, which is used to mechanically remove plaque. The mechanical action facilitates the penetration of the freshly released BITC into deeper structures. Our findings of a strong bactericidal effect on Gram-negative bacteria but a minor effect on Gram-positives suggest a possible role for Miswak extract in oral hygiene odontological prophylaxis. We have previously demonstrated that rinsing with Miswak extracts raises the dental plaque pH [20]. However, the biofilm life-style within dental plaque makes bacteria generally less susceptible to chemical agents and *in vivo* studies will be required to assess the effect of Miswak and Miswak extract on the composition of the plaque microflora.

Naturally occurring isothiocyanates are released during consumption of cruciferous vegetables [39]. These compounds have been demonstrated to possess cancer-preventive activity in animal models [40] and a high dietary intake of isothiocyanates is associated with a reduced risk of cancer in humans [41], [42]. Some studies have addressed the possible adverse effects of BITC but the *in vivo* tolerance is high compared to *in vitro* effects on cell-lines [43], [44] indicating that BITC is detoxified in the living organism [45]. It is therefore unlikely that BITC release upon use of chewing sticks from *S. persica* would cause adverse reactions in the oral cavity or in the gut.

In conclusion; BITC is the main antibacterial component of *S. persica* root chewing sticks with high killing activity against the Gram-negative periodontal pathogens *A. actinomycetemcomitans* and *P. gingivalis.* Extracts from *S persica* root might thus be an avenue to explore for applications as an adjunct to treatment of periodontal diseases. The strong and rapid killing affected exclusively Gram-negative bacteria, including medically important pathogens such as *Salmonella enterica, Pseudomonas aeroginosa* and *Haemophilus influenza*. The results advocate in favour of further investigating the Gram-negative specificity of BITC as a potential antimicrobial substance for therapeutic use.

## Supporting Information

Figure S1 **Thin-layer chromatography of MPLC-fractions.**
(PDF)

Table S1 Retardation factor (Rf) of MPLC fractions determined by TLC analysis.
(PDF)
