# Supplementary material for: Benzyl Isothiocyanate, a Major Component from the Roots of Salvadora Persica Is Highly Active against Gram-Negative Bacteria
Source: PLoS One. 2011 Aug 1;6(8):e23045. doi: 10.1371/journal.pone.0023045 (PMC3148225; doi:10.1371/journal.pone.0023045)

Figure S1: Thin-layer chromatography of MPLC-fractions
Mobile phase is in **(A)** 10% ethyl acetate in hexane, **(B)** 20% ethyl acetate in hexane and in **(C)** 30% methanol in ethyl acetate. Numbers denote MPLC-fraction number. The MPLC fractions were pooled into eight samples, marked by arrows in the figure. The fractions were pooled on the basis of the retardation factors of the different compounds (see Table S1). Fractions 1-7 (sample 1), fractions 8-18 (sample 2), fractions 19-20 (sample 3), fractions 21-23 (sample 4), fractions 24-30 (sample 5), fractions 31-53 (sample 6), fractions 54-56 (sample 7) and fractions 57-70 (sample 8).

A

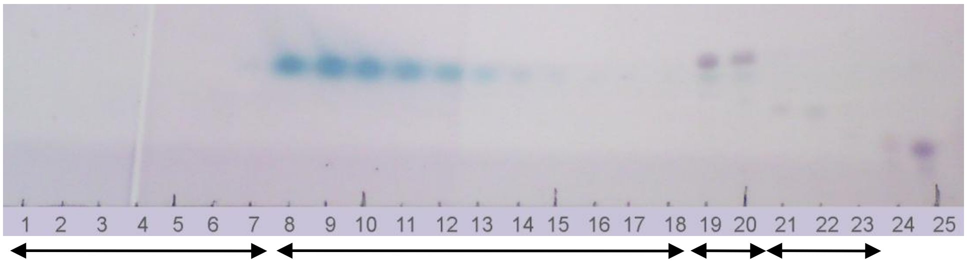

B

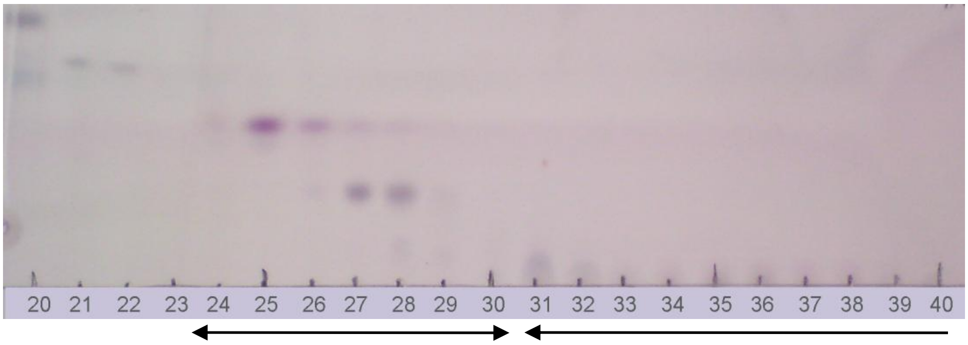

C

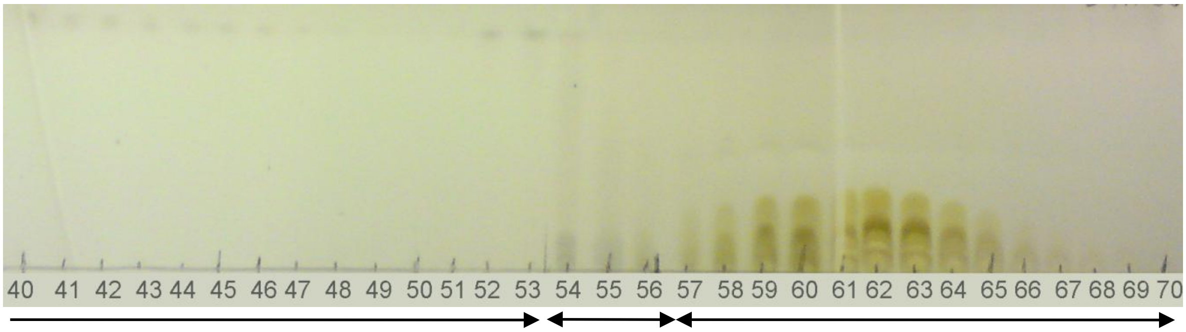

Supplement: Figure S1 — Thin-layer chromatography of MPLC-fractions. (PDF) [file pone.0023045.s001.pdf]
