# Supplementary material for: Benzyl Isothiocyanate, a Major Component from the Roots of Salvadora Persica Is Highly Active against Gram-Negative Bacteria
Source: PLoS One. 2011 Aug 1;6(8):e23045. doi: 10.1371/journal.pone.0023045 (PMC3148225; doi:10.1371/journal.pone.0023045)
Supplement: Table S1 — Retardation factor (Rf) of MPLC fractions determined by TLC analysis. (PDF) [file pone.0023045.s002.pdf]

**Table S1. Retardation factor (Rf) of MPLC fractions determined by TLC analysis**

| **MPLC fraction number *** | **Rf** | **Pooled MPLC sample** |
|---|---|---|
| 1-7 | 1.0 | 1 |
| 8-18 | 0.4 | 2 |
| 19-20 | 0.4-0.5 | 3 |
| 21-23 | 0.3-0.4 | 4 |
| 24-30 | 0.2 | 5 |
| 31-53 | 0.1 | 6 |
| 54-56 | >0.1 | 7 |
| 57-70 | >0.1 | 8 |

*MPLC fraction number refers to supplemental Figure S1.
